# TWIST1 promotes invasion through mesenchymal change in human glioblastoma

**DOI:** 10.1186/1476-4598-9-194

**Published:** 2010-07-20

**Authors:** Svetlana A Mikheeva, Andrei M Mikheev, Audrey Petit, Richard Beyer, Robert G Oxford, Leila Khorasani, John-Patrick Maxwell, Carlotta A Glackin, Hiroaki Wakimoto, Inés González-Herrero, Isidro Sánchez-García, John R Silber, Philip J Horner, Robert C Rostomily

**Affiliations:** 1 Department of Neurological Surgery, University of Washington School of Medicine, Seattle WA, 98195, USA; 2 Environmental Health Sciences, University of Washington School of Medicine, Seattle WA, 98195, USA; 3 Institute for Stem Cell and Regenerative Medicine, University of Washington School of Medicine, Seattle WA, 98195 USA; 4 Division of Molecular Medicine, Beckman Research Institute of the City of Hope, Duarte CA, 91010, USA; 5 Molecular Neurosurgery Laboratory, Massachusetts General Hospital, Harvard Medical School, Boston MA, 02114 USA; 6 Experimental Therapeutics and Translational Oncology Program, Instituto de Biología Molecular y Celular del Cáncer (IBMCC), CSIC/Universidad de Salamanca, Campus Unamuno, 37007-Salamanca, Spain; 7 Petrov Research Institute of Oncology, St.-Petersburg, 197758, Russia

## Abstract

**Background:**

Tumor cell invasion into adjacent normal brain is a mesenchymal feature of GBM and a major factor contributing to their dismal outcomes. Therefore, better understandings of mechanisms that promote mesenchymal change in GBM are of great clinical importance to address invasion. We previously showed that the bHLH transcription factor TWIST1 which orchestrates carcinoma metastasis through an epithelial mesenchymal transition (EMT) is upregulated in GBM and promotes invasion of the SF767 GBM cell line *in vitro*.

**Results:**

To further define TWIST1 functions in GBM we tested the impact of TWIST1 over-expression on invasion *in vivo *and its impact on gene expression. We found that TWIST1 significantly increased SNB19 and T98G cell line invasion in orthotopic xenotransplants and increased expression of genes in functional categories associated with adhesion, extracellular matrix proteins, cell motility and locomotion, cell migration and actin cytoskeleton organization. Consistent with this TWIST1 reduced cell aggregation, promoted actin cytoskeletal re-organization and enhanced migration and adhesion to fibronectin substrates. Individual genes upregulated by TWIST1 known to promote EMT and/or GBM invasion included SNAI2, MMP2, HGF, FAP and FN1. Distinct from carcinoma EMT, TWIST1 did not generate an E- to N-cadherin "switch" in GBM cell lines. The clinical relevance of putative TWIST target genes SNAI2 and fibroblast activation protein alpha (FAP) identified *in vitro *was confirmed by their highly correlated expression with TWIST1 in 39 human tumors. The potential therapeutic importance of inhibiting TWIST1 was also shown through a decrease in cell invasion *in vitro *and growth of GBM stem cells.

**Conclusions:**

Together these studies demonstrated that TWIST1 enhances GBM invasion in concert with mesenchymal change not involving the canonical cadherin switch of carcinoma EMT. Given the recent recognition that mesenchymal change in GBMs is associated with increased malignancy, these findings support the potential therapeutic importance of strategies to subvert TWIST1-mediated mesenchymal change.

## Background

Invasion is arguably the feature of human glioblastoma (GBM) most responsible for their dismal outcomes with average survival less than 1 year. Diffuse tumor invasion into adjacent brain restricts curative resection and limits effective delivery of chemotherapy and radiation. In addition, migratory GBM cells can activate mechanisms that increase resistance to these therapies further compounding efforts to eradicate them. Despite the importance of glioma invasion, little is known about how this complex phenotype is regulated in gliomas, a prerequisite to development of effective anti-invasion therapies. By contrast, the process by which human epithelial cancers, or carcinomas, acquire an invasive phenotype has been more extensively characterized at both the cellular and molecular levels.

Carcinoma invasion and metastasis are driven by a process termed epithelial to mesenchymal transition (EMT) (For review see [1]). Mesenchymal transitions lead to acquired potential for cell migration, changes in cytoskeletal organization, reduced cellular adhesion and changes in expression of transcription factors. Among the transcription factors that play fundamental roles in regulating these changes is the basic helix-loop-helix protein TWIST1. TWIST1 activates EMT in the context of embryonic morphogenesis [2], tissue fibrosis [3,4] and cancer metastasis [5-7]. A central feature of TWIST1-mediated EMT is the repression of the epithelial marker E-cadherin, and activation of the mesenchymal marker N-cadherin [5-7], a hallmark feature of carcinoma EMT termed the "cadherin switch". The recent recognition of mesenchymal change in glioblastoma [8-10] and its association with more aggressive clinical phenotypes [8,9] suggests that mechanisms that promote EMT in carcinoma may be of great clinical relevance in GBM.

We previously reported that TWIST1 is up-regulated in malignant gliomas and promotes glioma cell invasion of the SF767 glioma cell line *in vitro *[11]. However, the role of TWIST1 in promoting glioma invasion has not been investigated in the context of the brain microenvironment or as a mediator of mesenchymal change as occurs in carcinomas. In addition, the identification and clinical relevance of putative TWIST1 target genes in GBMs is not known. In this study we report that TWIST1 promoted GBM invasion through activation of mesenchymal molecular and cellular changes. This effect was not dependent on a "cadherin switch" indicating that TWIST1 promotes invasion through mesenchymal changes distinct from those associated with carcinoma EMT. The highly correlated expression of TWIST1 and mesenchymal target genes SNAI2 and FAP in human gliomas supported the clinical relevance of TWIST1 mesenchymal change. Together these results demonstrated an important role of TWIST1 in glioma invasion through activation of mesenchymal change and suggest its potential as a therapeutic target.

## Results

### TWIST1 regulates invasion of multiple GBM cell lines *in vitro*

To extend our previous observation that TWIST1 enhanced invasion of the SF767 GBM cell line *in vitro *[11], we studied the effects of TWIST1 over-expression on invasion of SNB19 and T98G GBM cells using matrigel transwell assays. Compared with controls, over-expression of TWIST1 in SNB19 cells (SNB19 TW) and T98G cells (T98G TW) resulted in an increase of invasion of 68% and 80%, respectively (Additional file 1). The pro-invasive function of TWIST1 was also confirmed in a well-characterized primary GBM stem cell line (GBM4) [12] where a five-fold increase in TWIST1 expression resulted in a 140% increase in invasion *in vitro *(Additional file 2). Together, these results solidly established the physiologic importance of TWIST1 for GBM invasion by demonstrating its uniformly pro-invasive function in multiple GBM cell lines *in vitro*.

### TWIST1 promotes GBM cell invasion in brain slice cultures and intact brain *in vivo*

**Figure 1:**
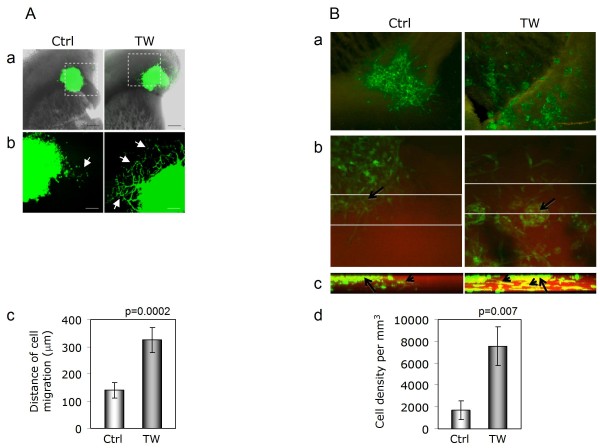
**TWIST1 over-expression increases invasion of SNB19 and T98G cells in organotypic brain slice**. **(A) **Representative low magnification (4×) images of SNB19 Ctrl and TWIST1 (TW) expressing cells cultured on the brain slices (a). Magnified sub-regions indicated by boxes in the top row are shown below (b). Cells invading brain tissue are shown with arrows. Scale bars: 500 and 200 μm. (c) Quantified cell migration is shown on the bar diagram (p = 0.0002). Brain slices were imaged using laser scanning confocal microscopy at equivalent optical planes and analyzed using Metamorph software. **(B) **(a): Low-magnification photomicrograph of merged fluorescent and DIC channel images. (Left) T98G control (Ctrl) cells generate defined cell aggregates in the brain slice surface. (Right) T98G cells with TWIST1 (TW) over-expression are dispersed as single cells and small non-cohesive aggregates over the brain slice. (b): Representative laser scanning confocal images of T98G Ctrl and T98G TW slices. Images obtained through the entire thickness of the brain slice and 50 μm sections (as indicated by white lines) were digitally reconstructed in the orthogonal plane for analysis of invasive cells. (c) Representative orthogonal views for T98G (Ctrl) and T98G TW are presented as a maximum-intensity projection image. Arrows show cell aggregates on the surface of the slice and arrowheads show invading cells. (d) The results of the analysis of invasive cell density demonstrated a significant increase in the invasion of T98G TW (7564 +/- 1771 cells/mm^3^) versus T98G control cells (1695 +/- 847 cells/mm^3^), p = 0.007.

To demonstrate pro-invasive TWIST1 function in more clinically relevant contexts, we characterized the growth patterns of SNB19 TW, T98G TW and corresponding control cells in an *ex vivo *model using organotypic brain slice cultures and *in vivo *using an orthotopic xenotransplant model. In *ex vivo *analyses, both SNB19 TW and T98G TW cells were significantly more invasive than control cells (Figure 1). Confocal microscopy confirmed that cells invaded into the brain slice rather than simply migrating along the surface. For SNB19, TWIST1 over-expression resulted in significantly increased distances of invasion compared with control cells when measured as orthogonal distances of cell migration from the border of the cohesive cell aggregate (p = 0.0002) (Figure 1A). T98G control cells placed on the brain slice formed a cohesive cell aggregate but T98G TW cells did not form a central cohesive core in 7 of 8 slices and diffused from the implant site into the brain slice as small cell clumps (Figure 1B). Therefore, for T98G we analyzed invasive cell density through the entire thickness of the brain slice using orthogonal reconstruction of confocal optical slices. Results shown in Figure 1B demonstrate that invading density of T98G TW cells is 4 fold higher compared to invading cell density of control cells.

**Figure 2:**
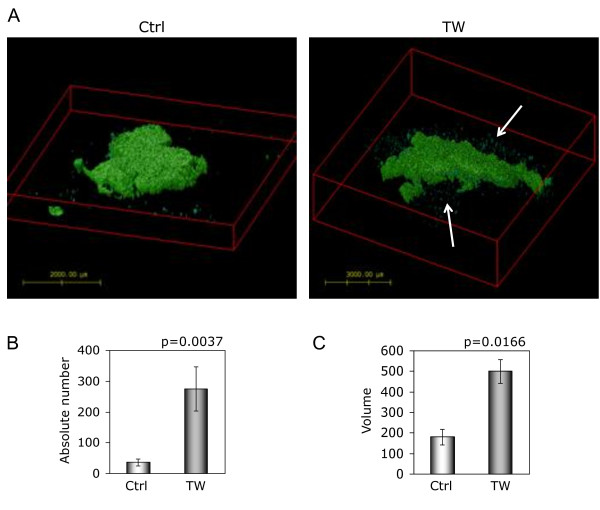
**TWIST1 over-expression increased invasiveness of SNB19 cells *in vivo***. **(A) **Representative images generated after intracranial injection of GFP-labeled SNB19 Ctrl (left) and SNB19 TW cells (right) show isolated and aggregate cell invasion adjacent to a central tumor core (type 2 growth pattern). Each 3-D confocal image is from a representative individual axial brain slice used for reconstruction of the growth pattern of the total tumor. After imaging and reconstruction, whole brain images were analyzed using Huygens software. The number of invasive aggregates **(B)**, and total volume (VoxVol) of invasive aggregates per tumor **(C) **were significantly greater in SNB19 TW cells compared with controls (p = 0.0037 and 0.0166, respectively). Arrows demonstrate invasive cell aggregates around central tumor core.

**Figure 3:**
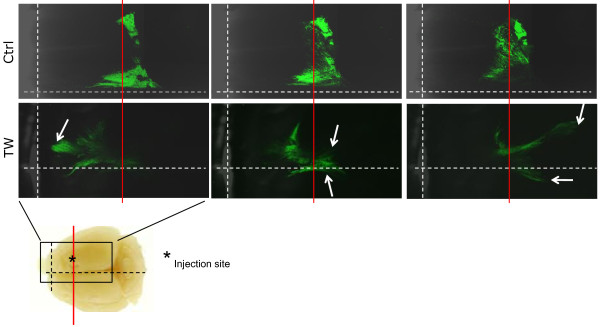
**TWIST1 over-expression increased invasiveness of T98G cells *in vivo***. Equal numbers of GFP-labeled T98G Ctrl and T98G TW cells were injected into SCID mouse brains. At three months, whole brains were isolated and imaged using laser scanning confocal microscopy. For each tumor three optical sections for corresponding levels within each tumor are shown (left -top of the tumor; center - middle of the tumor; right - bottom of the tumor). Dashed white vertical and horizontal lines correspond to anterior border of brain and midline, respectively. The solid red line corresponds to the fixed coronal plane through the site of injection. Whole brain picture (lower left panel) demonstrates site of injection and approximate borders of the confocal images. In contrast to the fixed position of tumor growth at the injection site for the T98G Ctrl tumor, the T98G TW tumor demonstrates a marked degree of tumor cell migration anterior and posterior to the plane of injection and across the midline.

**Figure 4:**
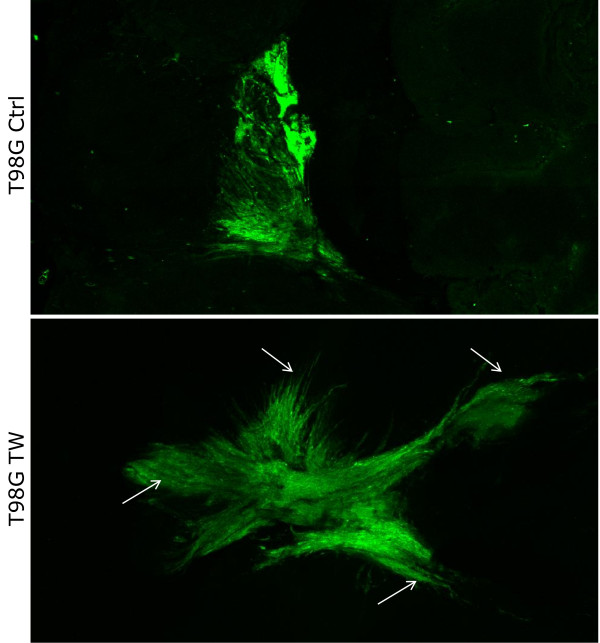
**Brightest point projection images (BPI) of tumor growth patterns**. The same tumors shown in Figure 3 were analyzed using BPI to visualize global differences in tumor growth patterns. Control T98G and T98G TWIST1 over-expressing tumor images are generated from total of 140 and 164 optical sections, respectively, collected by confocal microscope using ImageJ software. Control tumors possess a more cohesive pattern and localized growth pattern while T98G TW tumors demonstrate a markedly diffuse pattern of growth (type 3 invasive growth pattern). Arrows indicate regions of diffuse T98G TW tumor cell outgrowth.

To determine whether TWIST1 over-expression increased invasion in the intact brain, we implanted SNB19 TW and T98G TW and corresponding control cells stably expressing GFP protein into the caudate nucleus of immuno-compromised adult mice (SNB19 TW (n = 6), control (n = 4); T98G TW and control (n = 3 each)). All animals were sacrificed when neurologic morbidity was apparent in the first animal (day 17 for SNB19 and day 90 for T98G). Whole brain fluorescent imaging by laser scanning confocal microscopy followed by computer-aided digital image reconstruction provided a comprehensive global comparison of the tumor growth patterns throughout the entire brain of each animal. SNB19 TW and control tumors all met criteria for Type 2 pattern of invasion with a central core surrounded by individual invasive cells and cell aggregates. Therefore, to determine differences in invasiveness between SNB19 TW and control tumors we used Huygens image analysis software to quantify the number of fluorescent particles detected as discrete objects separate from the tumor core (indicating migratory tumor cells or clumps), the composite volume of these particles for each tumor and finally the core tumor volumes. A representative reconstructed tumor slice volume showing cores and invasive particles is shown in Figure 2A. This analysis demonstrated a significant increase in the number of discrete aggregates separate from the tumor cores and the volume of the invasive particles in tumors with TWIST1 over-expression compared to control tumors (p = 0.0037 and p = 0.0166, respectively) (Figure 2B, C) and a trend towards larger mean tumor core volume in SNB19 TW tumors (p = 0.11). All T98G control tumors grew as localized expansile masses (Type 1 growth pattern) while all T98G TW tumors generated markedly invasive tumors that disseminated diffusely throughout the brain (Type 3 growth pattern) (Figures 3 and 4). Individual optical sections from corresponding areas of T98G control and TWIST1 tumors (Figure 3) and a brightest point projection image (BPI) (Figure 4) which sums the individual signals through the entire Z-stack onto a 2-dimensional image, demonstrated the differences in growth patterns. The growth pattern for all tumors generated from T98G TW cells appeared to coincide with white matter tracts and crossed midline (Figure 3), both recognized primary routes of human GBM tumor cell invasion. Taken together these results provided critical confirmation of the pro-invasive function of TWIST1 in GBM cells in the context of the brain microenvironment and demonstrated cell-type- specific variability in TWIST1-mediated patterns of invasion.

### TWIST1 activates cell type-specific gene expression profiles consistent with invasion

**Figure 5:**
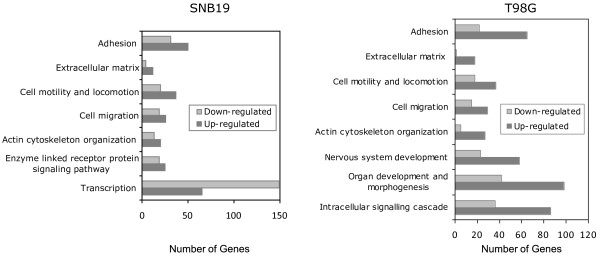
**TWIST1 over-expression results in over-representation of common GO categories related to mesenchymal phenotype and invasion**. To characterize changes in gene expression due to TWIST1 over-expression, we compared global gene expression in vector control and TWIST1 over-expressing SNB19 and T98G cells using the Affymetrix GeneChip platform. The analysis revealed many common categories identified by GoMiner (FDR cut-off level <0.1) that are related to mesenchymal function and invasion (cell adhesion, extracellular matrix, cell migration, cell motility and locomotion, and actin cytoskeleton organization) as well as cell-line specific categories consistent with TWIST1 function (transcription and organ development and morphogenesis, e.g.). The number of up- and down-regulated genes in each category is shown. The individual genes that comprise each category are provided in Additional file 4, Table S1.

To characterize the molecular basis of TWIST1 expression and associated pro-invasive phenotypes we compared expression profiles of SNB19 and T98G cells with TWIST1 over-expression and corresponding control cells using Affymetrix gene expression arrays. Overall, TWIST1 over-expression led to differential regulation of 1924 genes in SNB19 and 1525 genes in T98G cell lines (1.5 fold, p < 0.05). Among these, 189 common genes were differentially regulated by TWIST1 over-expression in both cell lines (Pearson's correlation 0.557; p < 0.05; Additional file 3). Using GO analysis we determined biological process gene categories that were significantly altered by TWIST1 over-expression (FDR = 0.1). In both cell lines TWIST1 over-expression resulted in consistent and significant over-representation of genes in GO biological process categories including cell adhesion, extracellular matrix, cell motility and locomotion, cell migration and actin cytoskeleton organization (Figure 5). Of note, these categories are fundamental biological processes that are component features collectively required to achieve cell invasion [1,13]. GO biological process categories unique to SNB19 included enzyme-linked receptors and transcription while T98G cell type-specific categories included development, morphogenesis and intracellular signaling cascade. Of interest, a significant over-representation of genes in nervous system development, a daughter category with the parent category of development, was also noted in T98G TW cells. The specific genes regulated by TWIST1 in these cell lines are discussed in more detail below. While this analysis demonstrated that cell type-specific changes in functional gene expression occur in GBM cells over-expressing TWIST1, we found that TWIST1 most consistently activated common molecular motifs related to cell invasion in both GBM cell lines.

### TWIST1 mediated alteration of cell-cell adhesion, cell-substrate interactions, migration, and actin cytoskeleton in glioma cells

**Figure 6:**
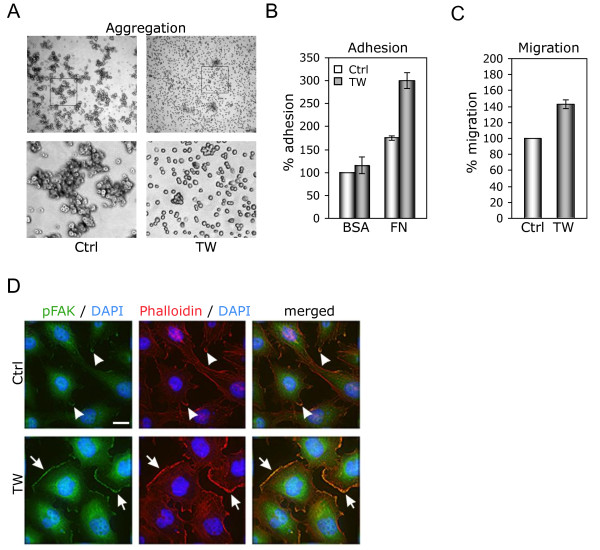
**TWIST1 over-expression produces mesenchymal cellular changes consistent with changes in gene expression**. GO analysis of gene expression predicted that TWIST1 would impact cellular phenotype and generate mesenchymal changes relevant to cell invasion. We tested this prediction in SNB19 cells using multiple cell-based assays. **(A) **TWIST1 inhibits cell aggregation. Representative (4×) image of cell aggregation of SNB19 Ctrl vs SNB19 TW cells (top row). Magnified (20×) sub-regions indicated by boxes in top row are shown below. **(B) **TWIST1 over-expression promoted adhesion to FN but not BSA-coated plates (shown as percent relative SNB19 control cells; mean ± SE). **(C) **Migration of SNB19 Tw cells through a filter membrane is increased 40% compared with SNB19 Ctrl cells. **(D) **Cell morphology, actin cytoskeleton architecture and FAK phosphorylation are altered by TWIST1. Representative (60×) photomicrographs of TWIST1 over-expressing and SNB19 control cells stained with anti-phospho-Tyr397 FAK (pFAK) antibodies (green), phalloidin-TRITC (red) and DAPI (blue). Phospho-FAK co-localization with F-actin along the border of lamellipodia in SNB19 TW and control cells is shown with large arrows and small arrows, respectively. Scale bar = 18 μm.

To determine whether the GO analysis was consistent with TWIST1-mediated cellular phenotypes we performed functional assays in SNB19 TW cells for changes in cell-cell adhesion, cell-substrate interactions, migration and actin cytoskeleton compared to control cells (SNB19 Ctrl) (Figure 6). SNB19 TW cells formed far fewer and smaller cellular aggregates compared with control cells indicating a significant change in cell-cell interactions (Figure 6A). Cell substrate interaction, tested by cell plating on fibronectin (FN) showed over 100% greater adhesion of SNB19 TW cells than control cells compared with no significant change found when cells are plated on bovine serum albumin (Figure 6B). Migration of SNB19 TW cells through an uncoated filter membrane increased 40% compared with controls (Figure 6C). Consistent with increased migration, SNB19 TW cells showed reorganization of actin cytoskeleton with increased lamellipodia formation associated with activation of focal adhesion kinase (FAK) at leading edges (Figure 6D). Together, these results demonstrated a concordant relationship between the TWIST1-mediated program of gene expression and cellular phenotypes. Furthermore these data indicated that TWIST1 regulates the multiple individual cellular changes (alteration of cell-cell interaction, cell-substrate interactions and reorganization of actin cytoskeleton to facilitate migration) that together comprise the carefully orchestrated process of glioma invasion.

### TWIST1 activates expression of mesenchymal genes without cadherin switch

**Figure 7:**
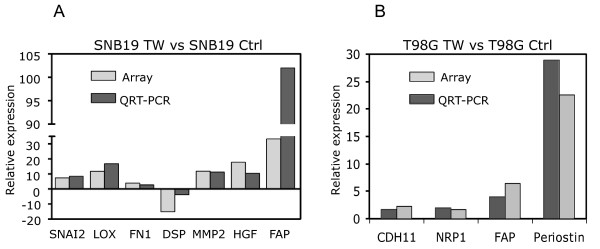
**Confirmation of the microarray results by qRT-PCR**. To validate the results from microarray experiments we compared the expression of selected genes related to EMT and/or carcinoma or GBM invasion identified in microarrays (see Tables 1 and Additional file 4, Table S1) with qRT-PCR from mRNA extracted from independent samples of SNB19 **(A) **and T98G **(B) **cells with and without TWIST1 over-expression.

The previous data demonstrated that TWIST1 over-expression promoted an invasive mesenchymal cellular phenotype in GBM cells. Given that TWIST1 promotes carcinoma invasion and metastasis through activation of EMT we sought to determine whether TWIST1 activated molecular features associated with carcinoma EMT in GBM cells. Within GO categories common to both SNB19 and T98G, approximately 1/3 of genes were upregulated in both cell lines while 2/3 were cell-type specific (Additional file 4, Table S1, S2). Many of these were genes known to promote mesenchymal changes in epithelial cancers (Table 1). Differential expression of a subset of genes selected from the arrays was validated by comparing expression in microarray with qRT-PCR (Figure 7). Genes associated with EMT were regulated by TWIST1 in SNB19 and/or T98G GBM cells including extracellular matrix proteins fibronectin 1 (FN1) [14], periostin (POSTN) [15] and SPARC [16-18] protease MMP2 [19,20], transcription factor SNAI2 [21-23] transcriptional modifier ID1 [24], growth factor HGF [21,25] lysyl oxidase (LOX) [26] and cell adhesion protein cadherin 11 (CDH11) [27]. Other genes associated with mesenchymal phenotypes and glioma invasion, not yet formally linked to EMT, included laminin, alpha 4 (LAMA4) [28] and fibroblast activation protein alpha (FAP) [29].

**Table 1:** EMT and invasion genes regulated by TWIST1 in SNB19 and/or T98G GBM cell lines

Gene symbol	Gene ID	Gene name	Fold differences	
			
			SNB19	T98G
SNAI2	213139_at	snail homolog 2	7.4	1
ID1	208937_s_at	inhibitor of DNA binding 1	2.0	2.1
ID2	213931_at	inhibitor of DNA binding 2	1.9	1.9
LOX	204298_s_at	lysyl oxidase	12	1
TIMP3	201149_s_at	TIMP metallopeptidase inhibitor 3	7.5	1
SPARC	212667_at	secreted protein, acidic, cysteine-rich	2.0	1
PDGFRB	202273_at	platelet-derived growth factor receptor	4.0	1
LAMA4	202202_s_at	laminin, alpha 4	7.7	4.8
FN1	216442_x_at	fibronectin 1	3.4	1
CDH11	236179_at	cadherin 11, type 2, OB-cadherin (osteoblast)	5.9	2.3
MMP2	201069_at	matrix metallopeptidase 2	12.7	1
FAP	209955_s_at	fibroblast activation protein, alpha	33.3	6.5
HGF	209960_at	hepatocyte growth factor	18	1
DSP	200606_at	Desmoplakin	-15.2	-3.8
IL8	211506_s_at	interleukin 8	7.5	12.1
POSTN	2105809_s_at	Periostin	1	22.8

**Figure 8:**
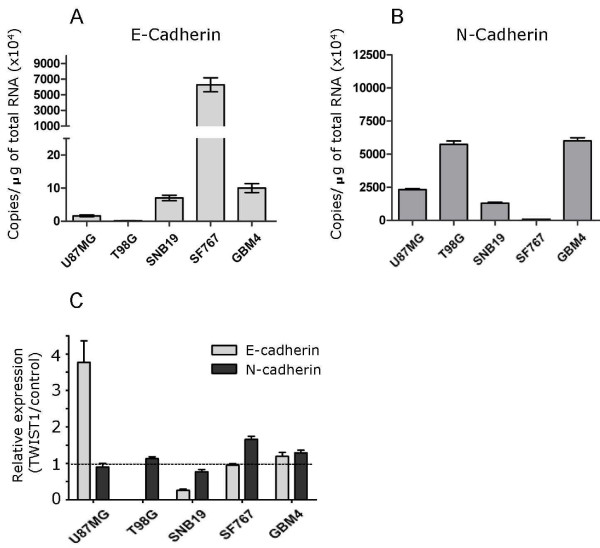
**TWIST1 over-expression does not generate an E- to N-cadherin switch in GBM cell lines**. Absolute quantification of E-cadherin **(A) **and N-cadherin **(B) **mRNA expression levels in GBM cells by qRT-PCR demonstrates variable levels of gene expression. Of note, E-cadherin is low or barely detectable in two of five lines tested. **(C) **Relative quantification of E- and N-cadherin gene expression in GBM cell lines with TWIST1 over-expression relative to control cells transduced with empty vector (accepted as 1 and shown as horizontal line). E-cadherin expression change is not shown for T98G because of expression levels close to background (see panel A). Contrary to carcinoma cells where TWIST1 expression activates a "cadherin switch" TWIST1 over-expression in GBM cell lines did not reduce E-cadherin expression concurrent with increased N-cadherin in any line tested.

Perhaps the most widely used marker for EMT in carcinomas is loss of E-cadherin and upregulation of N-cadherin, or the "cadherin switch". To determine whether TWIST1 activated a "cadherin switch" in GBM cells, we quantified E-cadherin and N-cadherin mRNA expression in a panel of GBM cell lines each with vector control or TWIST1 over-expression. We found no consistent relationship between endogenous levels of E- or N-cadherin or changes with TWIST1 over-expression (Figure 8). Of importance, in no case did TWIST1 over-expression result in combined reduction of E-cadherin and upregulation of N-cadherin. These data indicated that the canonical "cadherin switch" central to TWIST1-mediated EMT in carcinomas does not occur in GBM cell lines over-expressing TWIST1 nor is it required to promote an invasive mesenchymal phenotype in human GBM cells. In only one of four cell lines (SNB19) in which TWIST1-mediated invasion was formally tested, did E-cadherin expression decrease with TWIST1 over-expression (Figure 8C). While TWIST1 over-expression resulted in decreased cell-cell adhesion in SNB19 in association with reduced E-cadherin expression, a similar decrease in cell-cell adhesion was noted for T98G TW cells versus vector controls (data not shown) despite the absence of detectable E-cadherin in control cells. Together these findings indicated that mechanisms independent of the E- to N-cadherin switch promote TWIST1-mediated GBM cell invasiveness. However, the commonality of genes regulated by TWIST1 in GBM cell lines and carcinoma metastasis suggested that the molecular program in GBM does partially overlap with that of TWIST1-mediated EMT in carcinomas.

### TWIST1 expression highly correlates with *in vitro *TWIST1 target genes in human glioma samples

**Figure 9:**
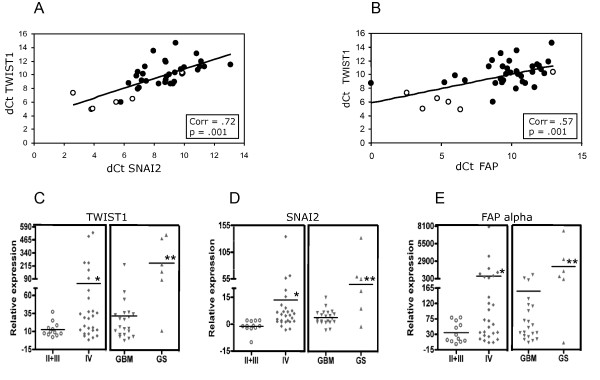
**Clinical relevance of putative TWIST1 *in vitro *target genes: SNAI2 and FAP were identified as putative TWIST1 target genes in SNB19 and/or T98G cells**. The expression of SNAI2 **(A) **and FAP **(B) **are highly correlated with TWIST1 expression in a group of 39 glial tumors of different grade and type including 9 grade II, 3 grade III and 27 grade IV (21 GBM, 6 gliosarcoma (GS) [open circles]) gliomas. ΔCt (dCt) values were determined by qRT-PCR. Correlation coefficients and p values are shown in insets. **(C, D, E) **The expression levels of TWIST1, SNAI2 and FAP relative to normal brain in different grades of glioma (II+III vs IV) and in the grade IV sub-types (GBM and gliosarcoma (GS)) are shown. Expression of TWIST1, SNAI2 and FAP are higher in grade IV gliomas (GBM and GS) compared to grades II and III combined (p = 0.022, p = 0.0014, p = 0.005, respectively). Analysis of grade IV tumors demonstrated higher levels of TWIST1, SNAI2 and FAP expression in GS compared to GBM (right side of each expression panel) (p = 0.0003, p = 0.0001, p = 0.0014, respectively). Horizontal bars show mean value of expression for each group of tumors. (*, ** p < 0.05). Statistical analysis was performed using log transformed relative expression values.

To determine whether expression of putative TWIST1 targets identified in microarrays are clinically relevant we compared expression of SNAI2 (upregulated 6.6 fold in SNB19 TW cells) and FAP (upregulated 36 fold in SNB19 and 6.5 fold inT98G). These genes were selected for study because of their characterized roles in carcinoma EMT invasion and metastasis [30] and their reported upregulation in malignant gliomas [31,32]. In a set of 39 human glial neoplasms using quantitative RT-PCR we found a significant correlation between TWIST1 and SNAI2 (r = 0.72; p = 0.001) and TWIST1 and FAP alpha (r = 0.57, p = 0.001) message levels (Figure 9A, B). Compared to normal brain controls, the mean TWIST1, SNAI2 and FAP expression were all up-regulated in the most malignant grade IV gliomas compared with grade II and III tumors (p = 0.022, p = 0.0014, p = 0.005, respectively) (Figure 9C, D, E). Consistent with their roles in mesodermal development and mesenchymal differentiation, the highest expression levels for all genes were evident in grade IV gliosarcoma tumors, which have the highest degree of overt mesenchymal differentiation among all gliomas. These results demonstrated for the first time a close association between expression of TWIST1 and putative TWIST1 target genes also implicated in both carcinoma and glioma malignancy. To confirm that putative TWIST1 targets play a role in glioma cell invasiveness, we overexpressed SNAI2 in SNB19 cells and found that SNAI2 was sufficient to increase SNB19 cell invasion 80% (Additional file 5). Together our results support the relevance of our *in vitro *model of TWIST1 function to identify candidate mechanisms of TWIST1-mediated invasion.

### Therapeutic significance of the inhibition of TWIST1 expression

**Figure 10:**
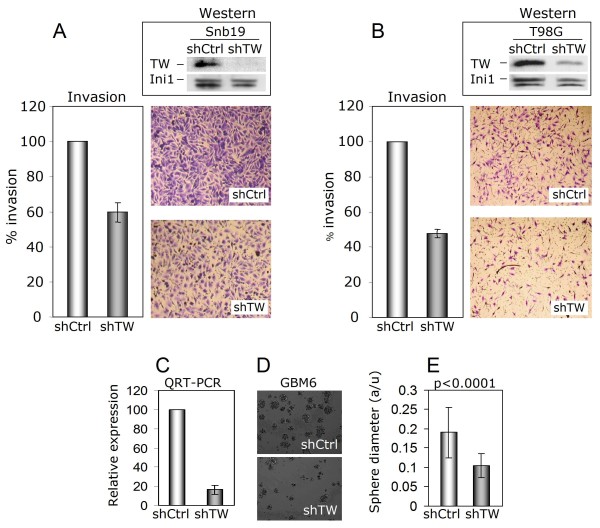
**Inhibition of TWIST1 expression decreases glioma cell invasion and stem cell activity**. **(A) **Top panel: Endogenous levels of TWIST1 protein expression in nuclear extracts from SNB19 cells transduced with shGFP (shCtrl) or shTWIST1 (shTW). Levels of Ini1 protein were used as a loading control for Western blot analysis. Bottom panel: Quantification of SNB19 shTW invasion relative to control cells (shCtrl) accepted as 100%. Representative images of membranes demonstrating reduced invasiveness of SNB19 shTW cells through matrigel relative to control cells are shown. **(B) **Endogenous levels of TWIST1 protein expression in nuclear extracts from T98G cells transduced with shGFP (shCtrl) or shTWIST1 (shTW). Levels of Ini1 protein were used as a loading control for Western blot analysis. Bottom panel: Quantification of T98G shTW invasion relative to control cells (shCtrl) accepted as 100%. Representative images of membranes demonstrating reduced invasiveness of T98G shTW cells through matrigel relative to control cells are shown. **(C) **Inhibition of TWIST1 mRNA expression by 80% was achieved in GBM6 glioma stem cells transduced with shTWIST1 resulting in reduced sphere size. **(D) **Phase contrast image of representative GBM6 spheres transduced with control or TWIST1 specific shRNA lentivirus shows dramatic qualitative reduction in sphere size (**E**) Quantification of mean sphere diameter in GBM6 control or shTW cells confirms significant decrease in sphere size due to inhibition of TWIST1 expression (p < 0.0001).

Our results demonstrated that increased levels of TWIST1 expression correlate with increased cell invasiveness. To determine whether inhibition of TWIST1 expression may have therapeutic relevance we investigated the effects of TWIST1 knockdown on cell invasion and glioma stem cell proliferation as well as tumor sphere formation using sequence-specific shRNA lentiviral constructs. In SNB19 and T98G cell lines, decreasing levels of TWIST1 message and protein resulted in inhibition of invasion. For SNB19, a 40% suppression of TWIST1 mRNA (not shown) and concurrent reduction in protein levels reduced invasion 53% (Figure 10A) while in T98G cells an 80% reduction of TWIST1 mRNA levels (not shown) and concurrent reduction in protein levels resulted in 40% inhibition of invasion through matrigel (Figure 10B). Invasion was compared to control cells stably infected with shGFP lentiviral constructs. These controls did not affect TWIST1 expression or invasion compared with the parental cells not infected with shGFP lentivirus (data not shown). A second, independent TWIST1-specific shRNA construct resulted in similar reductions of TWIST1 mRNA and invasion (data not shown). Finally, to demonstrate the specificity of the TWIST1 shRNAs we quantified their effects on the highly related gene DERMO1/TWIST2 and found no changes in its expression (data not shown). Using well-characterized glioma stem cells [12] we then tested the effect of TWIST1 inhibition on sphere-forming activity. Sphere number measures the frequency of cells capable of proliferating to form cell clusters while sphere size reflects the growth potential of each sphere. With 80% inhibition of TWIST1 expression in GBM6 stem cells, mean sphere size was reduced 48% (p < 0.0001) (Figure 10 C-E). In the GBM8 tumor stem cell line, similar 80% inhibition of TWIST1 message (data not shown) resulted in a marked decrease in the number of wells that formed tumor spheres when plated at clonal dilution (30% vs. 67% in control GBM8 cells; p = 0.0033) and the mean number of spheres in each well containing spheres (1.0 vs 2.3 for control GBM8 cells; p = 0.002) (see Table 2). Together these results indicated the potential therapeutic relevance of TWIST1 inhibition for invasion and abrogation of glioma stem cell properties.

**Table 2:** TWIST1 knockdown reduces sphere-forming activity of GBM8 stem cells

Cell type	Percent wells with spheres	Number of spheres/well+/-SE
shCTRL	67	2.3+/-0.3
shTW	30*	1+/-0.1**

* p = 0.0033; ** p = 0.002

## Discussion

The fundamental role for mesenchymal change in promoting invasion, malignancy treatment response and even cancer stem cell function in human carcinoma and GBM invasion is increasingly recognized [8,9]. TWIST1 is a central regulator of mesenchymal change in carcinoma [5] but its relevance to invasion and mesenchymal change in GBM models has not been studied. Since tumor invasion is perhaps the major obstacle to improved outcome for patients with carcinomas and gliomas the elucidation of TWIST1 function in GBMs is potentially of great clinical importance. Following on our previous observation in the SF767 GBM cell line [11] this study validated the pro-invasive function of TWIST1 in multiple cell lines *in vitro *and *in vivo *and demonstrated that TWIST1 promoted clinically relevant mesenchymal molecular and cellular phenotypes that partially recapitulated those associated with carcinoma EMT. These findings identify TWIST1 as a regulator of mesenchymal change and invasion in GBM that can be leveraged for further investigation of the clinical potential of subverting mesenchymal change as a therapeutic strategy in treating GBM.

Collectively TWIST1 promoted invasion *in vitro *of all GBM cells tested to date (including a GBM stem cell line). We further established that TWIST1 enhanced invasion in the more relevant settings of brain slice culture and orthotopic xenotransplant models using SNB19 and T98G GBM cell lines. Of interest, the patterns of enhanced invasion generated by TWIST1 over-expression were cell-line specific with SNB19 TW cells invading as single cells or small aggregates from a central core while T98G TW cells diffusely invaded throughout the brain. These extreme patterns of invasion are similar to those in cases of gliomatosis cerebri [33]. These findings clearly demonstrate the generic pro-invasive function for TWIST1 in GBM and suggest that cell-intrinsic factors can modify TWIST1- mediated patterns of GBM invasion.

Consistent with this, TWIST1 over-expression generated cell-specific changes in gene expression with shared pro-invasive functional attributes. TWIST1-mediated changes in expression of specific genes in SNB19 and T98G were heterogeneous but overlapped at the functional level within five common categories related to the cellular requirements for glioma invasion and EMT including cell adhesion, extracellular matrix, cell motility and locomotion, cell migration and actin cytoskeleton organization. Importantly, TWIST1 over-expression generated cell phenotypes highly consistent with the over-representation of genes within these functional categories that reflect critical individual cellular features required for carcinoma and GBM invasion [1]. We also determined that TWIST1 induced re-localization of activated FAK to sites of abundant lamellipodia formation, a significant finding given the association between FAK activation, cytoskeletal organization and its role in EMT and glioma malignancy (reviewed in [34-36]. Loss of apical-basal polarity (relative to a basement membrane) is an additional feature of EMT in carcinomas which was not tested here since assays of polarity for GBM cells *in vitro *are not well established. However, the recent description of polarized ciliated neural stem cells within the ventricular zone neuroepithelium [37-39] suggests that such studies could be attempted *in situ *or with novel co-culture systems or at earlier stages of glioma development. This approach could reveal polarity changes (analogous to carcinoma EMT) as fundamental steps in the process of gliomagenesis and acquisition of an invasive phenotype. Together gene expression analysis and cellular assays demonstrated that TWIST1 over-expression in glioma cells orchestrated the acquisition of a robust mesenchymal phenotype and cellular changes that closely mirror those of carcinoma cells undergoing mesenchymal transformation [40] and required for tumor invasion and metastasis [41].

TWIST1-mediated molecular changes also provided important insight into its role in mesenchymal change in GBM. Many genes related to carcinoma EMT were also up-regulated by TWIST1 in GBM indicating potential mechanistic overlap between the two processes. However, the lack of a TWIST1-mediated "cadherin switch" in GBM cells suggested that alternative mechanisms in nervous tissue and gliomas function to modulate cell adhesion and invasion. Alternatively, a cadherin switch could occur early in gliomagenesis or require specific anatomic or environmental interactions not present in our experimental system. The recent discovery that normal neural stem cells -- putative GBM cells of origin -- express E-cadherin supports this possibility [42,43]. Further studies are warranted to examine the impact of TWIST1 and other factors related to mesenchymal change in normal GBM cells of origin (neural stem and progenitor cells) or in cells at early stages of gliomagenesis to better define how alterations in E-cadherin or other cell-cell adhesion molecules impact the acquisition of an invasive malignant phenotype.

The clinical relevance of identified putative TWIST1 targets was established through correlation between TWIST1, SNAI2 and FAP expression levels in 39 human gliomas of different grades. These studies demonstrated that the current *in vitro *model of TWIST1 pro-invasive function was capable of identifying clinically relevant pro-invasive targets and candidate downstream mechanisms of TWIST1-mediated glioma invasion. Our data also confirms prior reports that expression of SNAI2 [31] and FAP [44] is directly linked to malignant glioma grade and further showed that they are coordinately upregulated in gliosarcoma, the grade IV glioma with the most overt mesenchymal differentiation. As regulators of invasiveness, TWIST1 and SNAI2 are potential targets for therapeutic modulation, a proposition further supported by their known functions to promote cell survival and treatment resistance in other cancer types [45-50]. FAP is expressed in wounds and fibrotic tissues as well as carcinoma-associated fibroblasts in multiple cancer types and is thought to degrade tumor matrix and facilitate carcinoma invasion [51]. Further studies are needed to determine which cell type(s) express FAP and whether it serves a similar role of altering tumor stroma to promote invasion in GBM.

The significance of TWIST1 function to promote invasion through mesenchymal change in GBMs is underscored by recent reports of clinically relevant mesenchymal phenotypes in GBMs. Gene expression array studies identified a mesenchymal stem cell (MSC) phenotype in human GBMs [10] and distinct pro-neural, proliferative and mesenchymal gene expression signatures among malignant grade III and IV human gliomas [9]. The mesenchymal signature is associated with poor prognosis, increased angiogenesis and tumor recurrence [9]. Therefore, along with other transcription factors such as STAT3 and C/EBP which were recently identified as regulators of mesenchymal transformation in GBM cells [8] the correlation of TWIST1 with induction of mesenchymal changes, increased glioma grade and invasiveness implicate TWIST1 as an additional central regulator of this process in human GBM. Of note, STAT3 transcriptionally upregulates TWIST1 expression and promotes breast carcinoma cell migration [52] prompting speculation that STAT3-TWIST1 interactions in GBM may also contribute to invasion and mesenchymal change.

Inhibitors of TWIST1 are not available; therefore, to investigate the therapeutic relevance of inhibiting TWIST1 in GBM we knocked down TWIST1 expression using shRNA and assayed its effects on cell invasion and glioma stem cell properties. Specific inhibition of TWIST expression resulted in marked reductions in glioma cell invasion *in vitro*. These findings are consistent with the pro-invasive function of TWIST1 in GBM and support the therapeutic potential of inhibiting TWIST1 or TWIST1-mediated signaling to inhibit GBM invasion. Glioma stem cells are recognized as tumor-initiating cells that determine tumor malignancy and growth. Through activation of EMT, TWIST1 promotes the formation and maintenance of breast cancer stem cells [53] and TWIST1 over-expression is implicated in mesenchymal stem cell activity [54]. Given these observations we propose that targeting TWIST1 may have additional therapeutic relevance in gliomas by abrogating glioma stem cell functions. Our data showed that inhibition of TWIST1 expression resulted in a dramatic reduction in GBM stem cell sphere formation and growth. These results suggest that a unique therapeutic potential of inhibiting TWIST1 may result from simultaneous targeting of glioma cell invasiveness and stem cell function -- hallmark GBM properties that both contribute to tumor growth, progression and treatment resistance. To address this potential, ongoing and future studies will address the effects of TWIST1 inhibition in GBM cells on tumor growth, invasion and response to therapy *in vivo*.

## Conclusions

Together these studies demonstrated that TWIST1 enhances GBM invasion in concert with mesenchymal changes. However, these changes do not involve the canonical cadherin switch of carcinoma EMT. The present findings demonstrated the potential usefulness of applying carcinoma EMT as a framework from which to enhance our understanding of GBM invasion and further suggest that a neural form of mesenchymal change, analogous to carcinoma EMT, may contribute broadly to glioma malignancy. Based on these findings we propose targeting TWIST1-mediated mesenchymal change as a therapeutic strategy with potential to inhibit GBM invasion and tumor growth, and enhance treatment responses.

## Methods

### Cell lines and tissue

Glioblastoma cell lines T98G, SNB19, SF767, U87MG were maintained in DMEM/F12 with 10% FBS (Hyclone). Human primary GBM cancer-initiated cells (GBM4, GBM6) were cultured as described [12] in the presence of EGF and bFGF. Human glioma tumor samples were acquired according to a protocol approved by the Institutional Review Board of the Human Subjects Division of the University of Washington. Samples were immediately snap frozen in liquid nitrogen and stored at -80°C before processing. Type and grade of tumors were confirmed by histopathological examination.

### Expression and shRNA constructs, and cell transduction

A retroviral human TWIST over-expression construct and methods for infection of SNB19 and T98G cell lines were described previously [11]. Myc-tagged SNAI2 expression construct (SNAI2myc) was generated by PCR followed by subcloning in LXSN expression vector. Exogenous protein expression was confirmed by Western blot analyses with corresponding antibody. Lentiviral shRNA construct for inhibition of TWIST1 expression [5] and control shRNA were purchased from Addgene. Lentivirus was generated using a standard method in HEK293T cells. A pool of infected cells was selected with Puromycin (1 μg/mL).

### Cell aggregation assay

Single-cell suspensions (10^5 ^cells/mL in DMEM-F12 without FBS) were plated into each well of a 6-well plate coated with 0.6% agarose/DMEM-F12. The plate was incubated at 37°C on a rocking platform for 16 hrs. Cells were fixed in 5% formalin to preserve cell-cell interactions and photographed.

### Cell adhesion

SNB19 LXSN and TWIST cells (5 × 10^4^/1 mL) were allowed to adhere to 24-well plates coated with BSA or fibronectin (5 μg/mL) for 1 hour. Cells were then washed, fixed, stained and counted. Differences in cell adhesion are shown as percent of SNB19 control cells attached to BSA-coated wells. Three wells from 3 separate experiments were analyzed and the significance of differences was determined by Student t-test. Data shown are mean ± SE.

### GBM stem cell sphere assays

GBM6 and GBM8 stem cells with TWIST1 knockdown and control cells (scrambled shRNA lentiviral vector) were dissociated and viable cells were counted using ViCell. Viable GBM6 cells plated at 3200 cells per well in 6-well plates were used to establish the effect of TWIST1 knockdown on sphere size. After 5 days spheres were photographed and sphere sizes were measured using Adobe Photoshop. To determine the effects of TWIST1 on sphere-forming activity GBM8 cells were plated at clonal dilution (20 viable cells per well in 96-well plate). After 7 days, wells with spheres were counted and presented as a percent of wells with spheres. Average numbers of spheres per well with spheres were also calculated. Fisher exact test and t-test were used for statistical analysis as appropriate.

### Immunocytochemistry and F-actin staining

Cells were grown in 8-well chambers and fixed in 4% PFA for 10 min. Following treatment with 0.1% Triton X-100/TBS for 5 min, cells were blocked in 1%BSA/TBS, washed and incubated with FAK or phospho-FAK antibody (Upstate) according to manufacturer protocol. Appropriate secondary antibody conjugated with FITC (Pierce) was used for antigen detection. F-actin was stained with TRITC conjugated phalloidin (1 μg/mL) followed by DAPI (1 μg/mL) staining. Antigens were visualized using confocal microscopy (Delta-Vision).

### Western blot analysis

Cell harvesting, cell lysis and Western blot procedures were performed as described previously [11]. Total cell lysates were used for detection of TWIST1 over-expression. Nuclear extracts were used to increase assay sensitivity in detecting endogenous TWIST1 expression. For protein loading control, anti-β-actin antibody (Sigma) for total proteins or Ini1 (H-300) antibody (Santa Cruz) for nuclear proteins were used. Immunoblot for MycTag Antibody (Upstate) was performed according to manufacturer's recommendation.

### Invasion and migration assays

The invasion and migration assays were performed using 24-well Matrigel invasion chambers or uncoated Control inserts (BD Biosciences) as previously described [11]. Briefly, cells were resuspended in a serum-free DMEM and loaded into inserts (5 × 10^4 ^cells/500 μL). DMEM/F-12 with 10% FBS (SNB19) and without FBS (T98G) was added to the lower chamber (750 μL). Following incubation at 37°C, cells that invaded or migrated to the underside of the membrane were fixed, stained, digitally imaged and counted. Differences in cell invasion were expressed as a percent of invading/migrating relative to control cells. Data shown are mean ± SE.

### Invasion in organotypic brain slices

Coronal brain slices (400 μm) from 21-day-old mice were cultured as previously described in the media supplemented with 10%FBS [55]. SNB19 or T98G cells with LXSN or TWIST1 over-expression (labeled with GFP expressing lentivirus) were placed at the corpus callosum. After 10 days *in vitro*, the co-cultures were fixed and analyzed by confocal microscopy. Slices were imaged using a FluoView FV1000 confocal microscope (Olympus). Collected Z-stacks were processed for visualization and cell counting using NIH Image software. The morphometric software Metamorph (Molecular Devices Corporation) was used to measure cell migration. The distances from the border of the cells' aggregate to each of the 20 furthest cells were measured for each of the 9 tissue slices (total n = 178 measurements) and compared using a repeated measures ANOVA model. The modeling was done with the 'proc mixed' procedure available in SAS.

### Intracranial injection and whole brain imaging

Animal experiments were performed according to procedures approved by the University of Washington IACUC. Glioma cells harboring empty vector and cells with TWIST1 over-expression were labeled with GFP-expressing lentivirus (pLL3.7) prior to implantation. T98G cells were injected in 9- to 10-week-old SCID-NOD mice. SNB19 cells were injected in 7- to 8-week-old nude mice. Following animal sedation 3 × 10^5 ^labeled cells were injected intracranially into the right caudate nucleus using a stereotactic apparatus and a Hamilton syringe. To determine the effect of TWIST1 on tumor growth and invasion, animals (6 mice with SNB19 TW and 4 mice with SNB19 Ctrl) were sacrificed 17 days after injection when the animals first showed signs of morbidity. Animals injected with T98G TW or control cells (3 mice per group) were sacrificed 90 days after injection. Animals were perfused with 4% paraformaldehyde (PFA). The entire brain was dissected from the calvarial vault and fixed for an additional 24 hours in 4% PFA at room temperature with light agitation. Brains were washed with PBS and transferred to 50% glycerol in PBS for 24 hours, 75% glycerol for 24 hours, then 90% glycerol at 4°C until imaged. The whole brain was sliced in the axial plane to obtain 3 slices, each approximately 2 mm thick. Each slice then was imaged using the FV1000 laser scanning confocal microscope in the axial plane to detect GFP-expressing cells and perform automated image splicing to reconstruct the entire tumor in a single axial slice.

### Image analysis for invasion

A semi-quantitative scale was used for initial characterization of tumor growth patterns as follows: Type 1 = solid compact core with mainly localized expansile growth; Type 2 = easily detectable non-contiguous individual cells or cell clusters invading into brain parenchyma adjacent to the solid tumor core; Type 3 = poorly defined or absent core with diffusely invasive contiguous or non-contiguous growth. Type 3 represents the most invasive growth pattern. For circumstances where the scale did not provide clear indications for the degree of invasiveness, differences in tumor growth pattern were quantified as follows: Reconstructed wide-field brain images with tumors were analyzed using Huygens software (Scientific Volume Imaging, Hilversum, The Netherlands). Individual signal intensities from each individual optical section (Z-stack images) collected by confocal microscopy were integrated into a brightest point projection image (BPI) to provide a 2-dimensional summary of total tumor cell density and spatial distributions (Image J). Information collected includes tumor core volume, invasive cell volume and a number of invasive aggregates. Numerical values were compared using t-test.

### Microarray processing methods for the Affymetrix microarray platform

Gene expression experiments were performed using the GeneChip platform by Affymetrix (Santa Clara, CA) and the manufacturer's protocol. Statistical analysis and data normalization for the Affymetrix arrays were carried out with Bioconductor software [56], and GeneTraffic^® ^(Iobion Informatics LLC, La Jolla, CA). Modified t-test was applied for two-group comparison. Bioconductor was used to calculate p-values using a modified t-test in conjunction with an empirical Bayes method to moderate the standard errors of the estimated log-fold changes. P-values were adjusted for multiplicity with the program q-value. Genes with absolute change greater than or equal to 1.5 fold and p < 0.05 were considered differentially regulated by TWIST1. To demonstrate the correlation of changes in expression (up or down-regulation) between genes differentially regulated in both cell lines, we applied a Pearson's correlation analysis using Bioconductor software (see above). Gene Ontology (GO) categories were analyzed using GoMiner [57] to detect gene category over-representation with cut off false discovery rate (FDR) 0.1.

### Quantitative RT-PCR (qRT-PCR)

Total RNA from cells, brain or tumor samples was extracted using Qiagen RNeasy mini kit. RNA (1 μg) was reverse-transcribed with Clontech kit. SYBR Green PCR Master mix (ABI) was used for template amplification. Thermocycling for all targets was carried out in 30 μL reaction for 40 cycles in triplicate. Each cycle consisted of: 94°C for 15 seconds, 58°C for 30 seconds and 72°C for 30 seconds. For all samples, reactions were run in triplicate. PCR reactions where reverse transcriptase was omitted were used as negative controls. SYBR Green incorporation was monitored in real time with an ABI PRISM 7000 sequence detection system (Applied Biosystems) and threshold exponential amplification cycle (C_T_) was calculated by SDS system software. Differences in the C_T _values (ΔC_T_) between the target transcript and GAPDH endogenous control determined the relative gene expression level and the ΔΔC_T _method was used to calculate fold differences in expression. Relative expression in each tumor sample is normalized by expression of corresponding target in a pool of normal brain samples (n = 4). Correlations between TWIST1 and SNAI2 or FAP expression were calculated using a regression coefficient and ΔCt values for each tumor sample. Statistical analysis of relative expression levels in human tumors was performed using unpaired t-test. Relative expression values were log transformed before testing to ensure normal distribution. Absolute quantification was used to compare levels of E- and N-cadherin mRNA expression. Standard curve was built using plasmid harboring corresponding cDNA targets diluted from 0 to 10^7 ^copies per reaction. Final results are shown as a number of copies per μg of total RNA. The specificity of amplifications was confirmed by amplicon melting profile.

## Abbreviations

EGF: epidermal growth factor; bFGF: basic fibroblast growth factor; FAP: fibroblast activating protein alpha; FAK: focal adhesion kinase; SNAI2: snail homolog 2; MMP2: Matrix metalloproteinase-2; HGF: hepatocyte growth factor; LAMA4: laminin alpha 4; LOX: lysyl oxidase; ADAM12: A disintegrin and metalloproteinase domain 12; DSP: desmoplakin; FBS: fetal bovine serum.

## Competing interests

The authors declare that they have no competing interests.

## Authors' contributions

SAM generated expression constructs and performed *in vitro *cellular assays, qRT-PCR and microarrays. AMM carried out *in vivo *studies, confocal imaging, microarray interpretation and statistical data analysis. AP performed *ex vivo *invasion assays. RB performed microarray analysis and bioinformatics support. RGO and LK participated in tumor collection and assisted with cell culture experiments. JPM participated in generation of recombinant DNAs and analysis of gene over-expression and knockdown. CAG generated anti-TWIST1 antibody. HW generated primary GBM cells. IG-H and IS-G generated reagents for Snai2 analysis. JRS and PJH assisted in experiment design, data interpretation and manuscript writing. RCR conceived of the study, and participated in its design, coordination and manuscript writing. All authors read and approved the final manuscript.

## Supplementary Material

Additional file 1 **Alteration of TWIST1 expression correlates with cell invasiveness *in vitro* in SNB19 and T98G cells.(A) **Top panel: Detection of TWIST1 protein from whole cell lysates of a pool of SNB19 cells transduced with retroviral expression construct (TW) compared to SNB19 cells transduced with empty vector (Ctrl). β-Actin is shown as loading control. Bottom panel: Quantification of SNB19 Tw cell invasion relative to Ctrl cells accepted as 100%. Representative images of membranes demonstrating increased invasiveness of Snb19 Tw cells relative to control cells are shown. **(B) **Top panel: Detection of exogenous TWIST1 over-expression in cell lysates from T98G cells by Western blot. Bottom panel: Quantification of T98G Tw cell invasion relative to Ctrl cells accepted as 100%. Representative images of membranes demonstrating increased invasiveness of T98G Tw cells relative to control cells are shown. Click here for file

Additional file 2 **Over-expression of TWIST1 expression in GBM stem cells correlates with cell invasiveness (A)** Quantification of exogenous TWIST1 over-expression using qRT-PCR in GBM4 primary GBM stem cells cultured as neurospheres and transduced with TWIST1 retroviral expression vector. **(B) **Quantification of GBM4 cell invasiveness in matrigel assay. Representative images of membranes demonstrating increased invasiveness of GBM4 Tw cells relative to control are shown. Differences in cell invasion are shown as percent of control cells transduced with empty vector (mean ± SE). Click here for file

Additional file 3 **Pearson's correlation of genes differentially regulated by TWIST1 in T98G and SNB19 cells with TWIST1 over-expression relative to corresponding controls**. A total of 189 genes (1.5 fold, p < 0.05) were differentially co-regulated by TWIST1 in both T98G and SNB19 cells relative to corresponding controls. Click here for file

Additional file 4 **Supplementary Tables**. **Table S1**. The list of common and cell-specific differentially expressed genes within each GO category over-represented in both SNB19 and T98G cells. **Table S2**. The total number of genes within common categories shown in Table S1. Click here for file

Additional file 5 **Putative TWIST1 target SNAI2 is sufficient to induce glioma invasiveness *in vitro***. **(A) **Exogenous over-expression of Myc-tagged SNAI2 in SNB19 cells. **(B) **Quantification of invasion of SNB19 cells with SNAI2 over-expression. Representative images of invasive cells on the membrane are shown. Click here for file
