# Supplementary material for: TWIST1 promotes invasion through mesenchymal change in human glioblastoma
Source: Mol Cancer. 2010 Jul 20;9:194. doi: 10.1186/1476-4598-9-194 (PMC2920263; doi:10.1186/1476-4598-9-194)
Supplement: Additional file 1 — Alteration of TWIST1 expression correlates with cell invasiveness in vitro in SNB19 and T98G cells.(A) Top panel: Detection of TWIST1 protein from whole cell lysates of a pool of SNB19 cells transduced with retroviral expression construct (TW) compared to SNB19 cells transduced with empty vector (Ctrl). β-Actin is shown as loading control. Bottom panel: Quantification of SNB19 Tw cell invasion relative to Ctrl cells accepted as 100%. Representative images of membranes demonstrating increased invasiveness of Snb19 Tw cells relative to control cells are shown. (B) Top panel: Detection of exogenous TWIST1 over-expression in cell lysates from T98G cells by Western blot. Bottom panel: Quantification of T98G Tw cell invasion relative to Ctrl cells accepted as 100%. Representative images of membranes demonstrating increased invasiveness of T98G Tw cells relative to control cells are shown. [file 1476-4598-9-194-S1.PPT]

## Slide 1
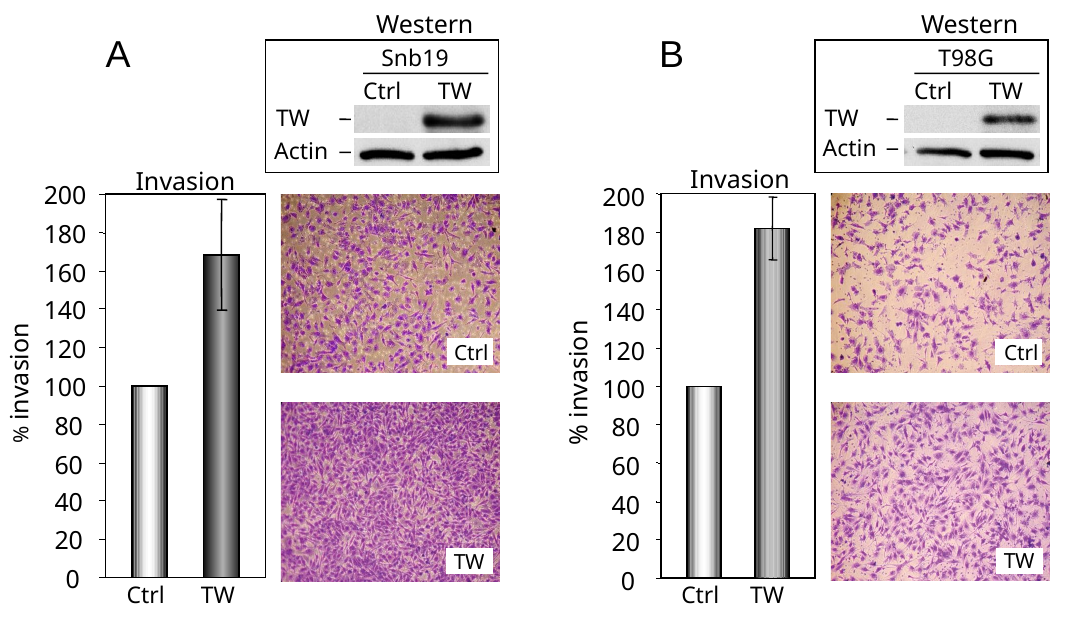

Western
Snb19
Ctrl
TW
TW
Actin
Invasion
200
180
160
140
120
100
80
60
40
20
0
Ctrl
% invasion
TW
Ctrl
TW
A
Western
T98G
Ctrl
TW
TW
Actin
Invasion
200
180
160
140
120
100
80
60
40
20
0
Ctrl
% invasion
TW
Ctrl
TW
B
