# Supplementary figures and images for: TWIST1 promotes invasion through mesenchymal change in human glioblastoma
Source: Mol Cancer. 2010 Jul 20;9:194. doi: 10.1186/1476-4598-9-194 (PMC2920263; doi:10.1186/1476-4598-9-194)

## Slide 1
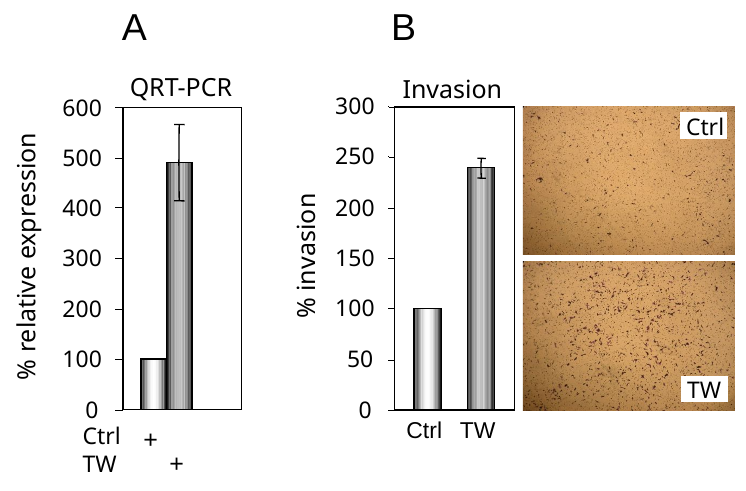

A
QRT-PCR
600
500
400
% relative expression
300
200
100
0
Ctrl
+
+
TW
B
Invasion
300
Ctrl
250
200
% invasion
150
100
50
TW
0
Ctrl
TW

Supplement: Additional file 2 — Over-expression of TWIST1 expression in GBM stem cells correlates with cell invasiveness (A) Quantification of exogenous TWIST1 over-expression using qRT-PCR in GBM4 primary GBM stem cells cultured as neurospheres and transduced with TWIST1 retroviral expression vector. (B) Quantification of GBM4 cell invasiveness in matrigel assay. Representative images of membranes demonstrating increased invasiveness of GBM4 Tw cells relative to control are shown. Differences in cell invasion are shown as percent of control cells transduced with empty vector (mean ± SE). [file 1476-4598-9-194-S2.PPT]

## Slide 1
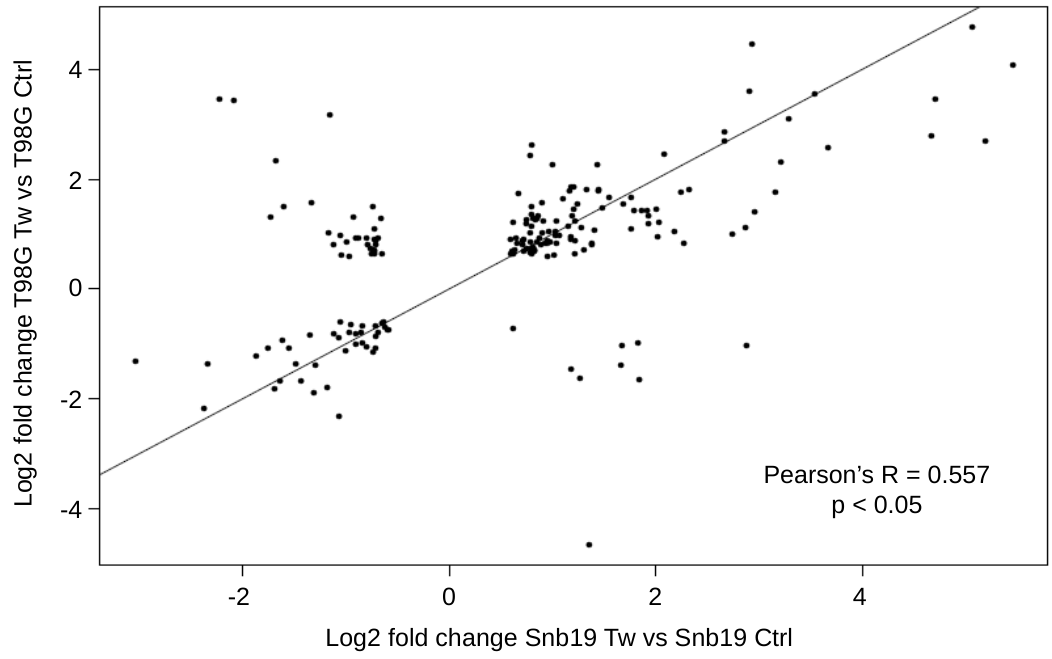

4
2
Log2 fold change T98G Tw vs T98G Ctrl
0
-2
Pearson’s R = 0.557
p < 0.05
-4
-2
0
2
4
Log2 fold change Snb19 Tw vs Snb19 Ctrl

Supplement: Additional file 3 — Pearson's correlation of genes differentially regulated by TWIST1 in T98G and SNB19 cells with TWIST1 over-expression relative to corresponding controls. A total of 189 genes (1.5 fold, p < 0.05) were differentially co-regulated by TWIST1 in both T98G and SNB19 cells relative to corresponding controls. [file 1476-4598-9-194-S3.PPT]

## Slide 1
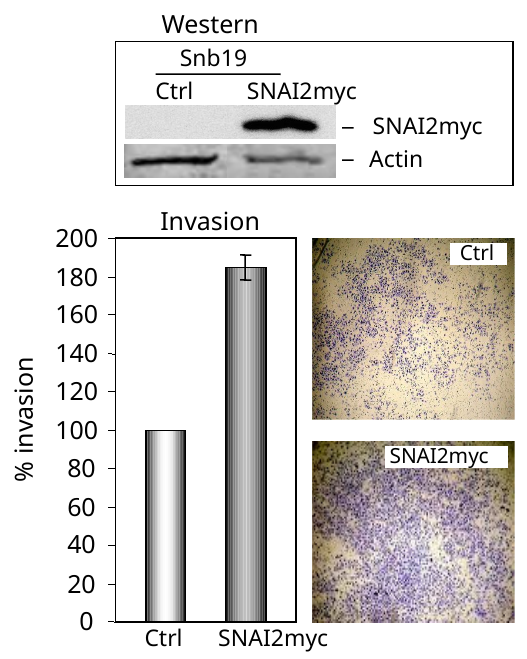

Western
Snb19
Ctrl
SNAI2myc
SNAI2myc
Actin
Invasion
200
Ctrl
180
160
140
120
% invasion
100
SNAI2myc
80
60
40
20
0
Ctrl
SNAI2myc

Supplement: Additional file 5 — Putative TWIST1 target SNAI2 is sufficient to induce glioma invasiveness in vitro. (A) Exogenous over-expression of Myc-tagged SNAI2 in SNB19 cells. (B) Quantification of invasion of SNB19 cells with SNAI2 over-expression. Representative images of invasive cells on the membrane are shown. [file 1476-4598-9-194-S5.PPT]
