# Supplementary material for: TWIST1 promotes invasion through mesenchymal change in human glioblastoma
Source: Mol Cancer. 2010 Jul 20;9:194. doi: 10.1186/1476-4598-9-194 (PMC2920263; doi:10.1186/1476-4598-9-194)
Supplement: Additional file 4 — Supplementary Tables. Table S1. The list of common and cell-specific differentially expressed genes within each GO category over-represented in both SNB19 and T98G cells. Table S2. The total number of genes within common categories shown in Table S1. [file 1476-4598-9-194-S4.PDF]

Table 1S **The list of up-regulated genes found within common GO catrgories over-represented (FDR<0.1) in cells with TWIST1 over-expression compared to control**

Gene symbol	Probe ID	Gene name	Fold up-regulation	
**Cell adhesion**				
Common genes			SNB19	T98G
APC	203526_s_at	adenomatosis polyposis coli	1.6	1.6
ARHGDIB	201288_at	Rho GDP dissociation inhibitor (GDI) beta	2.1	2.1
CCL2	216598_s_at	chemokine (C-C motif) ligand 2	44.0	16.7
CDH11	215608_at	cadherin 11, type 2, OB-cadherin (osteoblast)	5.9	2.3
COL6A1	213428_s_at	collagen, type VI, alpha 1	2.9	2.2
COL6A2	209156_s_at	collagen, type VI, alpha 2	8.9	3.4
COL7A1	204136_at	collagen, type VII, alpha 1 (epidermolysis bullosa, dystrophic, dominant and recessive)	2.8	1.6
DPT	213071_at	dermatopontin	4.2	5.4
ECE1	201749_at	endothelin converting enzyme 1	1.6	1.6
GSN	214040_s_at	gelsolin (amyloidosis, Finnish type)	2.7	1.8
IL8	211506_s_at	interleukin 8	7.5	12.1
ITGA5	201389_at	integrin, alpha 5 (fibronectin receptor, alpha polypeptide)	1.7	2.6
KIFAP3	203333_at	kinesin-associated protein 3	1.6	1.9
LAMA4	202202_s_at	laminin, alpha 4	7.7	4.8
NRP1	212298_at	neuropilin 1	2.6	1.7
PCDHGC3	215836_s_at	protocadherin gamma subfamily C, 3	3.5	2.3
SDC1	201287_s_at	syndecan 1	1.8	1.7
SRPX	204955_at	sushi-repeat-containing protein, X-linked	5.0	3.5
TGFBI	201506_at	transforming growth factor, beta-induced, 68kDa	2.5	1.6
THY1	213869_x_at	Thy-1 cell surface antigen	1.7	2.2
TNFAIP6	206026_s_at	tumor necrosis factor, alpha-induced protein 6	6.3	5.9
ZYX	215706_x_at	zyxin	1.5	1.6
SNB19 specific genes				
CAPS	226424_at	calcyphosine	1.7	
CAT	201432_at	catalase	2.1	
CD44	204489_s_at	CD44 molecule (Indian blood group)	1.7	
COL12A1	225664_at	collagen, type XII, alpha 1	2.3	
COL5A2	221729_at	collagen, type V, alpha 2	4.3	
DLC1	220512_at	deleted in liver cancer 1	2.1	
DLG5	201681_s_at	discs, large homolog 5 (Drosophila)	1.7	
FBLIM1	225258_at	filamin binding LIM protein 1	1.7	
FBLN5	203088_at	fibulin 5	4.6	
FN1	212464_s_at	fibronectin 1	3.4	
ITGBL1	214927_at	integrin, beta-like 1 (with EGF-like repeat domains)	2.5	
JUP	201015_s_at	junction plakoglobin	2.1	

LMO7	242722_at	LIM domain 7	1.7	
LOX	204298_s_at	lysyl oxidase	12.0	
LOXL2	202998_s_at	lysyl oxidase-like 2	1.7	
LPP	202822_at	LIM domain containing preferred translocation partner in lipoma	1.6	
MMD	203414_at	monocyte to macrophage differentiation-associated	3.2	
NRM	225592_at	nurim (nuclear envelope membrane protein)	1.9	
PCDH10	228635_at	protocadherin 10	8.3	
PCDH18	225977_at	protocadherin 18	5.5	
PCDH7	205534_at	BH-protocadherin (brain-heart)	3.0	
PDPN	226658_at	podoplanin	2.0	
PKD1	202328_s_at	polycystic kidney disease 1 (autosomal dominant)	1.6	
PTPRF	215066_at	protein tyrosine phosphatase, receptor type, F	2.5	
SPARC	212667_at	secreted protein, acidic, cysteine-rich (osteonectin)	2.0	
THBS2	203083_at	thrombospondin 2	10.8	
TPBG	203476_at	trophoblast glycoprotein	3.4	
TSPAN5	209890_at	tetraspanin 5	4.6	
T98G specific genes				
ADAM12	213790_at	ADAM metallopeptidase domain 12 (meltrin alpha)		3.5
APLP1	209462_at	amyloid beta (A4) precursor-like protein 1		1.8
APP	211277_x_at	amyloid beta (A4) precursor protein (peptidase nexin-II, Alzheimer disease)		1.6
ARMC8	203487_s_at	armadillo repeat containing 8		1.7
CD58	216942_s_at	CD58 molecule		1.6
CDH4	220227_at	cadherin 4, type 1, R-cadherin (retinal)		3.9
COL13A1	211343_s_at	collagen, type XIII, alpha 1		5.7
COL1A1	202310_s_at	collagen, type I, alpha 1		4.8
COL1A2	229218_at	collagen, type I, alpha 2		2.3
COL27A1	225293_at	collagen, type XXVII, alpha 1		2.4
COL4A1	211981_at	collagen, type IV, alpha 1		2.5
COL4A2	211966_at	collagen, type IV, alpha 2		2.1
COL5A1	212489_at	collagen, type V, alpha 1		1.9
CSPG2	221731_x_at	chondroitin sulfate proteoglycan 2 (versican)		2.6
CXCL12	209687_at	chemokine (C-X-C motif) ligand 12 (stromal cell-derived factor 1)		3.6
DLG1	217208_s_at	discs, large homolog 1 (Drosophila)		1.5
EFS	204400_at	embryonal Fyn-associated substrate		2.0
FXYD5	224252_s_at	FXYD domain containing ion transport regulator 5		1.7
HES1	203395_s_at	hairy and enhancer of split 1, (Drosophila)		2.2
HNT	227566_at	neurotrimin		9.6
ITGA11	222899_at	integrin, alpha 11		1.5
ITGB1	1553678_a_at	integrin, beta 1 (fibronectin receptor, beta polypeptide, antigen CD29 includes MDF2, MSK12)		1.9
KITLG	226534_at	KIT ligand		2.5
LAMB2	216264_s_at	laminin, beta 2 (laminin S)		3.8
LAMB3	209270_at	laminin, beta 3		2.6
LAMC1	200771_at	laminin, gamma 1 (formerly LAMB2)		1.6

LPXN	216250_s_at	leupaxin		26.8
MKLN1	204423_at	muskelin 1, intracellular mediator containing kelch motifs		1.6
MPDZ	205079_s_at	multiple PDZ domain protein		1.7
NID1	202008_s_at	nidogen 1		2.5
PARVA	222454_s_at	parvin, alpha		1.6
PCDHB14	231726_at	protocadherin beta 14		2.5
PCDHB16	232099_at	protocadherin beta 16		3.7
PCDHGA1	209079_x_at	protocadherin gamma subfamily A, 1		2.5
PCDHGA11	211876_x_at	protocadherin gamma subfamily A, 11		3.2
PCDHGA3	216352_x_at	protocadherin gamma subfamily A, 3		3.6
POSTN	210809_s_at	periostin, osteoblast specific factor		22.8
PPFIBP1	203736_s_at	PTPRF interacting protein, binding protein 1 (liprin beta 1)		1.9
SEMA5A	205405_at	sema domain, seven thrombospondin repeats (type 1 and type 1-like), transmembrane5A domain (TM) and short cytoplasmic domain, (semaphorin)		1.7
SIRPA	202897_at	signal-regulatory protein alpha		2.1
SPOCK1	202363_at	sparc/osteonectin, cwcv and kazal-like domains proteoglycan (testican) 1		5.1
SSX2IP	203019_x_at	synovial sarcoma, X breakpoint 2 interacting protein		2.5
WISP1	211312_s_at	WNT1 inducible signaling pathway protein 1		6.0
**Extracellular matrix**				
Common genes			*SNB19*	*T98G*
ADAM19	209765_at	ADAM metallopeptidase domain 19 (meltrin beta)	1.9	2.0
COL6A1	213428_s_at	collagen, type VI, alpha 1	2.9	2.2
COL6A2	209156_s_at	collagen, type VI, alpha 2	8.9	3.4
COL7A1	204136_at	collagen, type VII, alpha 1 (epidermolysis bullosa, dystrophic, dominant and recessive)	2.8	1.6
DPT	213071_at	dermatopontin	4.2	5.4
FBLN1	202995_s_at	fibulin 1	2.5	1.8
FBN1	235318_at	fibrillin 1	2.4	2.3
FBN2	203184_at	fibrillin 2 (congenital contractural arachnodactyly)	2.0	2.5
LAMA4	202202_s_at	laminin, alpha 4	7.7	4.8
TGFBI	201506_at	transforming growth factor, beta-induced, 68kDa	2.5	1.6
SNB19 specific genes				
ADAMTS5	219935_at	ADAM metallopeptidase with thrombospondin type 1 motif, 5 (aggrecanase-2)	6.7	
COL12A1	225664_at	collagen, type XII, alpha 1	2.3	
COL5A2	221729_at	collagen, type V, alpha 2	4.3	
DCN	211896_s_at	decorin	1.9	
ECM1	209365_s_at	extracellular matrix protein 1	2.5	
FBLN5	203088_at	fibulin 5	4.6	
FN1	212464_s_at	fibronectin 1	3.4	
LGALS3	208949_s_at	lectin, galactoside-binding, soluble, 3 (galectin 3)	2.1	
LOX	204298_s_at	lysyl oxidase	12.0	
LPL	203549_s_at	lipoprotein lipase	6.3	

LTBP1	202729_s_at	latent transforming growth factor beta binding protein 1	6.6	
LUM	201744_s_at	lumican	7.8	
MMP2	201069_at	matrix metallopeptidase 2 (gelatinase A, 72kDa gelatinase, 72kDa type IV collagenase)	12.7	
NTNG2	233072_at	netrin G2	1.7	
PKD1	202328_s_at	polycystic kidney disease 1 (autosomal dominant)	1.6	
SNTB2	205315_s_at	syntrophin, beta 2 (dystrophin-associated protein A1, 59kDa, basic component 2)	1.8	
SPARC	212667_at	secreted protein, acidic, cysteine-rich (osteonectin)	2.0	
TGFBR3	226625_at	transforming growth factor, beta receptor III (betaglycan, 300kDa)	4.6	
THBS2	203083_at	thrombospondin 2	10.8	
TIMP2	224560_at	TIMP metallopeptidase inhibitor 2	1.7	
TIMP3	201149_s_at	TIMP metallopeptidase inhibitor 3 (Sorsby fundus dystrophy, pseudoinflammatory)	7.5	
TPBG	203476_at	trophoblast glycoprotein	3.4	
T98G specific genes				
ADAM12	213790_at	ADAM metallopeptidase domain 12 (meltrin alpha)		3.5
APLP1	209462_at	amyloid beta (A4) precursor-like protein 1		1.8
COL1A1	202310_s_at	collagen, type I, alpha 1		4.8
COL1A2	229218_at	collagen, type I, alpha 2		2.3
COL27A1	225293_at	collagen, type XXVII, alpha 1		2.4
COL4A1	211981_at	collagen, type IV, alpha 1		2.5
COL4A2	211966_at	collagen, type IV, alpha 2		2.1
COL5A1	212489_at	collagen, type V, alpha 1		1.9
CPZ	211062_s_at	carboxypeptidase Z		1.5
CSPG2	221731_x_at	chondroitin sulfate proteoglycan 2 (versican)		2.6
CTHRC1	225681_at	collagen triple helix repeat containing 1		3.3
LAMB2	216264_s_at	laminin, beta 2 (laminin S)		3.8
LAMB3	209270_at	laminin, beta 3		2.6
LAMC1	200771_at	laminin, gamma 1 (formerly LAMB2)		1.6
LTBP3	219922_s_at	latent transforming growth factor beta binding protein 3		1.8
MMP14	202828_s_at	matrix metallopeptidase 14 (membrane-inserted)		1.8
MMP16	208167_s_at	matrix metallopeptidase 16 (membrane-inserted)		4.2
NID1	202008_s_at	nidogen 1		2.5
POSTN	210809_s_at	periostin, osteoblast specific factor		22.8
SPOCK1	202363_at	sparc/osteonectin, cwcv and kazal-like domains proteoglycan (testican) 1		5.1
SYN2	243879_at	synapsin II		3.8
TIMP4	206243_at	TIMP metallopeptidase inhibitor 4		4.9
Cell motility and locomotion				
Common genes			*SNB19*	*T98G*
ARHGDIB	201288_at	Rho GDP dissociation inhibitor (GDI) beta	2.1	2.1
DPYSL3	201431_s_at	dihydropyrimidinase-like 3	4.5	2.1
IL8	211506_s_at	interleukin 8	7.5	12.1
LAMA4	202202_s_at	laminin, alpha 4	7.7	4.8
NAV1	227584_at	neuron navigator 1	1.7	1.6

NRP1	212298_at	neuropilin 1	2.6	1.7
SCG2	204035_at	secretogranin II (chromogranin C)	25.4	6.9
THY1	213869_x_at	Thy-1 cell surface antigen	1.7	2.2
VEGF	212171_x_at	vascular endothelial growth factor	1.9	2.2
SNB19 specific genes				
ANXA1	201012_at	annexin A1	1.7	
B3GALT6	225733_at	UDP-Gal:betaGal beta 1,3-galactosyltransferase polypeptide 6	2.4	
BTG1	200921_s_at	B-cell translocation gene 1, anti-proliferative	1.7	
CABP4	1554201_at	calcium binding protein 4	1.6	
CAP1	213798_s_at	CAP, adenylate cyclase-associated protein 1 (yeast)	1.5	
CAPS	226424_at	calcyphosine	1.7	
CAPZB	201950_x_at	capping protein (actin filament) muscle Z-line, beta	1.7	
CENTD3	218950_at	centaurin, delta 3	1.5	
CLIC4	221881_s_at	chloride intracellular channel 4	1.6	
CLU	222043_at	clusterin	1.8	
EFNB3	205031_at	ephrin-B3	5.7	
ETV1	217061_s_at	ets variant gene 1	1.8	
ETV4	211603_s_at	ets variant gene 4 (E1A enhancer binding protein, E1AF)	2.7	
FGFR1	210973_s_at	fibroblast growth factor receptor 1 (fms-related tyrosine kinase 2, Pfeiffer syndrome)	2.0	
FN1	212464_s_at	fibronectin 1	3.4	
FPR1	205119_s_at	formyl peptide receptor 1	4.1	
HGF	209961_s_at	hepatocyte growth factor (hepapoietin A; scatter factor)	18.0	
IFT57	222519_s_at	intraflagellar transport 57 homolog (Chlamydomonas)	2.1	
LEF1	221558_s_at	lymphoid enhancer-binding factor 1	3.3	
MYLIP	220319_s_at	myosin regulatory light chain interacting protein	1.6	
NR2F1	209505_at	nuclear receptor subfamily 2, group F, member 1	1.7	
PDPN	226658_at	podoplanin	2.0	
PTGES	207388_s_at	prostaglandin E synthase	2.2	
PTGS2	204748_at	prostaglandin-endoperoxide synthase 2 (prostaglandin G/H synthase and cyclooxygenase)	2.8	
S100A2	204268_at	S100 calcium binding protein A2	4.2	
TGFA	211258_s_at	transforming growth factor, alpha	1.9	
TPBG	203476_at	trophoblast glycoprotein	3.4	
TSPAN5	209890_at	tetraspanin 5	4.6	
T98G specific genes				
APP	211277_x_at	amyloid beta (A4) precursor protein (peptidase nexin-II, Alzheimer disease)		1.6
CCL25	206988_at	chemokine (C-C motif) ligand 25		2.0
CDH4	220227_at	cadherin 4, type 1, R-cadherin (retinal)		3.9
CNN2	201605_x_at	calponin 2		1.6
CSPG2	221731_x_at	chondroitin sulfate proteoglycan 2 (versican)		2.6
CTHRC1	225681_at	collagen triple helix repeat containing 1		3.3
CXCL12	209687_at	chemokine (C-X-C motif) ligand 12 (stromal cell-derived factor 1)		3.6
DNER	226281_at	delta/notch-like EGF repeat containing		10.7

DOCK2	235236_at	dedicator of cytokinesis 2		9.2
DVL3	201908_at	dishevelled, dsh homolog 3 (Drosophila)		2.0
FYN	216033_s_at	FYN oncogene related to SRC, FGR, YES		2.9
IL1B	39402_at	interleukin 1, beta		8.9
INSR	227432_s_at	insulin receptor		2.2
ITGA11	222899_at	integrin, alpha 11		1.5
ITGB1	1553678_a_at	integrin, beta 1 (fibronectin receptor, beta polypeptide, antigen CD29 includes MDF2, MSK12)		1.9
JAG1	216268_s_at	jagged 1 (Alagille syndrome)		3.2
LHX2	206140_at	LIM homeobox 2		1.9
MAPK8	226046_at	mitogen-activated protein kinase 8		1.8
MKLN1	204423_at	muskelin 1, intracellular mediator containing kelch motifs		1.6
PRKCI	209677_at	protein kinase C, iota		2.0
SEMA5A	205405_at	sema domain, seven thrombospondin repeats (type 1 and type 1-like), transmembrane 5A domain (TM) and short cytoplasmic domain, (semaphorin)		1.7
SIRPA	202897_at	signal-regulatory protein alpha		2.1
SPOCK1	202363_at	sparc/osteonectin, cwcv and kazal-like domains proteoglycan (testican) 1		5.1
TLN1	203254_s_at	talin 1		1.7
TSPAN6	209109_s_at	tetraspanin 6		1.9
UNC5B	226899_at	unc-5 homolog B (C. elegans)		2.4
VEGFC	209946_at	vascular endothelial growth factor C		1.8
WNT5B	223537_s_at	wingless-type MMTV integration site family, member 5B		2.0
**Cell migration**				
Common genes			*SNB19*	*T98G*
DPYSL3	201431_s_at	dihydropyrimidinase-like 3	4.5	2.1
IL8	211506_s_at	interleukin 8	7.5	12.1
LAMA4	202202_s_at	laminin, alpha 4	7.7	4.8
NAV1	227584_at	neuron navigator 1	1.7	1.6
NRP1	212298_at	neuropilin 1	2.6	1.7
SCG2	204035_at	secretogranin II (chromogranin C)	25.4	6.9
THY1	213869_x_at	Thy-1 cell surface antigen	1.7	2.2
VEGF	212171_x_at	vascular endothelial growth factor	1.9	2.2
SNB19 specific genes				
B3GALT6	225733_at	UDP-Gal:betaGal beta 1,3-galactosyltransferase polypeptide 6	2.4	
BTG1	200921_s_at	B-cell translocation gene 1, anti-proliferative	1.7	
CAP1	213798_s_at	CAP, adenylate cyclase-associated protein 1 (yeast)	1.5	
CAPS	226424_at	calcyphosine	1.7	
CENTD3	218950_at	centaurin, delta 3	1.5	
CLIC4	221881_s_at	chloride intracellular channel 4	1.6	
CLU	222043_at	clusterin	1.8	
EFNB3	205031_at	ephrin-B3	5.7	
ETV1	217061_s_at	ets variant gene 1	1.8	

ETV4	211603_s_at	ets variant gene 4 (E1A enhancer binding protein, E1AF)	2.7	
FGFR1	210973_s_at	fibroblast growth factor receptor 1 (fms-related tyrosine kinase 2, Pfeiffer syndrome)	2.0	
FN1	212464_s_at	fibronectin 1	3.4	
LEF1	221558_s_at	lymphoid enhancer-binding factor 1	3.3	
NR2F1	209505_at	nuclear receptor subfamily 2, group F, member 1	1.7	
PDPN	226658_at	podoplanin	2.0	
PTGES	207388_s_at	prostaglandin E synthase	2.2	
S100A2	204268_at	S100 calcium binding protein A2	4.2	
TGFA	211258_s_at	transforming growth factor, alpha	1.9	
T98G specific genes				
APP	211277_x_at	amyloid beta (A4) precursor protein (peptidase nexin-II, Alzheimer disease)		1.6
CCL25	206988_at	chemokine (C-C motif) ligand 25		2.0
CDH4	220227_at	cadherin 4, type 1, R-cadherin (retinal)		3.9
CNN2	201605_x_at	calponin 2		1.6
CTHRC1	225681_at	collagen triple helix repeat containing 1		3.3
CXCL12	209687_at	chemokine (C-X-C motif) ligand 12 (stromal cell-derived factor 1)		3.6
DNER	226281_at	delta/notch-like EGF repeat containing		10.7
DOCK2	235236_at	dedicator of cytokinesis 2		9.2
DVL3	201908_at	dishevelled, dsh homolog 3 (Drosophila)		2.0
FYN	216033_s_at	FYN oncogene related to SRC, FGR, YES		2.9
IL1B	39402_at	interleukin 1, beta		8.9
INSR	227432_s_at	insulin receptor		2.2
ITGA11	222899_at	integrin, alpha 11		1.5
ITGB1	1553678_a_at	integrin, beta 1 (fibronectin receptor, beta polypeptide, antigen CD29 includes MDF2, MSK12)		1.9
JAG1	216268_s_at	jagged 1 (Alagille syndrome)		3.2
LHX2	206140_at	LIM homeobox 2		1.9
PRKCI	209677_at	protein kinase C, iota		2.0
SEMA5A	205405_at	sema domain, seven thrombospondin repeats (type 1 and type 1-like), transmembrane 5A domain (TM) and short cytoplasmic domain, (semaphorin)		1.7
UNC5B	226899_at	unc-5 homolog B (C. elegans)		2.4
VEGFC	209946_at	vascular endothelial growth factor C		1.8
WNT5B	223537_s_at	wingless-type MMTV integration site family, member 5B		2.0
**Actin organization**				
Common genes			*SNB19*	*T98G*
ARHGDIB	201288_at	Rho GDP dissociation inhibitor (GDI) beta	2.1	2.1
DAAM1	216060_s_at	dishevelled associated activator of morphogenesis 1	2.1	3.1
EHD2	45297_at	EH-domain containing 2	1.8	1.6
GSN	214040_s_at	gelsolin (amyloidosis, Finnish type)	2.7	1.8
PDE4DIP	212390_at	phosphodiesterase 4D interacting protein (myomegalin)	1.7	1.9
RAC2	213603_s_at	ras-related C3 botulinum toxin substrate 2 (rho family, small GTP binding protein Rac2)	1.8	1.8
TRIOBP	216210_x_at	TRIO and F-actin binding protein	1.5	1.6

SNB19 specific genes				
ARHGEF2	1554783_s_at	rho/rac guanine nucleotide exchange factor (GEF) 2	2.2	
CAP1	213798_s_at	CAP, adenylate cyclase-associated protein 1 (yeast)	1.5	
CAPG	201850_at	capping protein (actin filament), gelsolin-like	2.1	
CAPZB	201950_x_at	capping protein (actin filament) muscle Z-line, beta	1.7	
DAAM2	212793_at	dishevelled associated activator of morphogenesis 2	7.9	
FBLIM1	225258_at	filamin binding LIM protein 1	1.7	
FSCN1	210933_s_at	fascin homolog 1, actin-bundling protein (Strongylocentrotus purpuratus)	2.0	
LMO7	242722_at	LIM domain 7	1.7	
SSH1	1555624_a_at	slingshot homolog 1 (Drosophila)	1.6	
WASF3	204042_at	WAS protein family, member 3	9.3	
WASPIP	202664_at	Wiskott-Aldrich syndrome protein interacting protein	2.0	
T98G specific genes				
AIM1	212543_at	absent in melanoma 1		2.0
CENTG1	1555907_at	centaurin, gamma 1		1.7
CNN2	201605_x_at	calponin 2		1.6
CXCL12	209687_at	chemokine (C-X-C motif) ligand 12 (stromal cell-derived factor 1)		3.6
DLG1	217208_s_at	discs, large homolog 1 (Drosophila)		1.5
DOCK2	235236_at	dedicator of cytokinesis 2		9.2
LCP1	208885_at	lymphocyte cytosolic protein 1 (L-plastin)		3.8
LLGL1	236594_at	lethal giant larvae homolog 1 (Drosophila)		1.7
MYO9B	217297_s_at	myosin IXB		1.6
NAPA	208751_at	N-ethylmaleimide-sensitive factor attachment protein, alpha		1.6
PARVA	222454_s_at	parvin, alpha		1.6
PDLIM7	214121_x_at	PDZ and LIM domain 7 (enigma)		1.6
PDPK1	244629_s_at	3-phosphoinositide dependent protein kinase-1		2.1
PRKCI	209677_at	protein kinase C, iota		2.0
SIRPA	202897_at	signal-regulatory protein alpha		2.1
SPTBN1	215918_s_at	spectrin, beta, non-erythrocytic 1		1.7
SSX2IP	203019_x_at	synovial sarcoma, X breakpoint 2 interacting protein		2.5
TLN1	203254_s_at	talin 1		1.7

Table 2S **The total number of genes within common categories shown in Table 1S**

	Common genes	SNB19 specific genes	T98G specific genes
Number of genes	35	71	76
